# Interplay between geo-population factors and hierarchy of cities in multilayer urban networks

**DOI:** 10.1038/s41598-017-17576-8

**Published:** 2017-12-08

**Authors:** Vladimir V. Makarov, Alexander E. Hramov, Daniil V. Kirsanov, Vladimir A. Maksimenko, Mikhail V. Goremyko, Alexey V. Ivanov, Ivan A. Yashkov, Stefano Boccaletti

**Affiliations:** 1 REC ‘Artificial Intelligence Systems and Neurotechnologies’, Yuri Gagarin State Technical University of Saratov, Polytechnicheskaja str 77, Saratov, 410054 Russia; 2 Institute of Energy and Transport Systems, Yurij Gagarin State Technical University of Saratov, Polytechnicheskaja str 77, Saratov, 410054 Russia; 3 Department of Automation, Control and Mechatronics, Yurij Gagarin State Technical University of Saratov, Polytechnicheskaja str 77, Saratov, 410054 Russia; 4 Institute of Urban Studies, Architecture and Construction, Yurij Gagarin State Technical University of Saratov, Polytechnicheskaja str 77, Saratov, 410054 Russia; 5 CNR-Institute of Complex Systems, Via Madonna del Piano 10, 50019 Sesto Fiorentino, Florence Italy; 6 The Italian Embassy in Israel, 25 Hamered Street, 68125 Tel Aviv, Israel

## Abstract

Only taking into consideration the interplay between processes occurring at different levels of a country can provide the complete social and geopolitical plot of its urban system. We study the interaction of the administrative structure and the geographical connectivity between cities with the help of a multiplex network approach. We found that a spatially-distributed geo-network imposes its own ranking to the hierarchical administrative network, while the latter redistributes the shortest paths between nodes in the geographical layer. Using both real demographic data of population censuses of the Republic of Kazakhstan and theoretical models, we show that in a country-scale urban network and for each specific city, the geographical neighbouring with highly populated areas is more important than its political setting. Furthermore, the structure of political subordination is instead crucial for the wealth of transportation network and communication between populated regions of the country.

## Introduction

Every process flowing in different parts of an urban system contributes to its economical and social outcome^1–3^. Therefore, attention is currently paid to analysis of the connectivity patterns of the networks which describe various layers of an urban complex. In^4^ authors study the ties between Chinese cities, that emerge themselves from the industrial relations, and found hierarchical tendencies in this network. Network-based approach is also used to describe and measure the public transport accessibility of different parts of Helsinki^5^. The work^6^ refers to socioeconomic relations between Mexico citizens, where homophily principles revealed in several aspects of this interactions. Public health status generally studied using network theory: in paper^7^ authors investigate the endemic transmission of HIV in Atlanta urban network. However, more and more frequently asked question is how these networks built on various criteria interact with each over in real world^8–10^. Recently, the multilayer networks’ approach has been introduced to properly represent the interaction structure between various aspects of urban networks, including social and cultural relationships^11,12^, health^13^, traffic flows^14,15^ and economics^16,17^. Although recent progresses on country-scale urban networks have been made^18,19^, in the majority of studies the application of a multiplex approach has been limited instead to the scale of a specific city or agglomeration.

Here we report on how the main features of spatially-distributed networks representing cities are directly affected by their hierarchical subordination within the larger scale of a country. For the first time, our study gives scientific ground and provides full understanding of two main phenomena: (i) in a country-scale urban network and for each specific city, the geographical neighbouring with highly populated areas is more important than its political setting, (ii) the structure of political subordination is crucial for the communication between populated regions of the country. Using both real data and theoretical models, we describe the fundamental aspects of interaction between spatially-distributed and spatially-independent connectivity structures, providing a new type of scale-free multilayer networks. Due to spatial constraints, this new structure does not exhibit direct connections between structural hubs (in contrast with the classical scale-free networks with high rich-club coefficients), which makes diffusion processes less effective, but can be compensated by interactions in the spatially-independent hierarchical layer. In particular, the emergence of inter-layer connections leads to the exchange of eigenvector and betweenness centralities of the nodes belonging to different layers.

The data, which we study, contains information gathered during population census of the Republic of Kazakhstan in seven time points, from 1939 to 2009. Each census includes the geographic coordinates and the number of citizens of all cities (and towns, the rural settlements are excluded) for the given year. The summary is presented in Table 1 and we should mention several facts emerging from the obtained data. First, one can notice the extremum in the number of cities, which falls on 1989, and this time point is also followed by the transfer of the capital from Alma-Ata to Astana. Actually, this year marks the beginning of reorganizing processes, which were most likely caused by the dissolution of the Soviet Union. The second apparent feature is a sharp decrease of the number of cities and a consistent growth of the average city population from 1999 to 2009. This reflects the strong outflow of people from towns to large cities, as Astana (the new capital) and Alma-Ata (the old capital, now city of republican significance). Other available public data allows to assume that such processes are still active, because in 2016 the population of Astana and Alma-Ata reached 1.0 and 1.7 millon people, respectively. The above-mentioned facts evidence, that the data reflects the various stages of evolution of an urban network of the country. This enables us to isolate the fundamental properties of such system and avoid the particular features of each stage of evolution.

**Table 1:** The summary of statistical data of urban network under study.

Year	Number of cities	Total population	Average population	Capital node	2^*nd*^ level nodes	3^*rd*^ level nodes	4^*th*^ level nodes
1939	83	1,710,027	20,603	Alma-Ata	20	43	19
1959	181	4,085,866	22,574	Alma-Ata	25	104	51
1970	254	6,538,652	25,743	Alma-Ata	26	142	84
1979	281	8,253,619	29,372	Alma-Ata	27	166	83
1989	291	9,276,510	31,769	Alma-Ata	27	174	90
1999	276	8,358,627	30,285	Astana	17	258	0
2009	139	8,909,333	64,096	Astana	15	123	0

**Figure 1:**
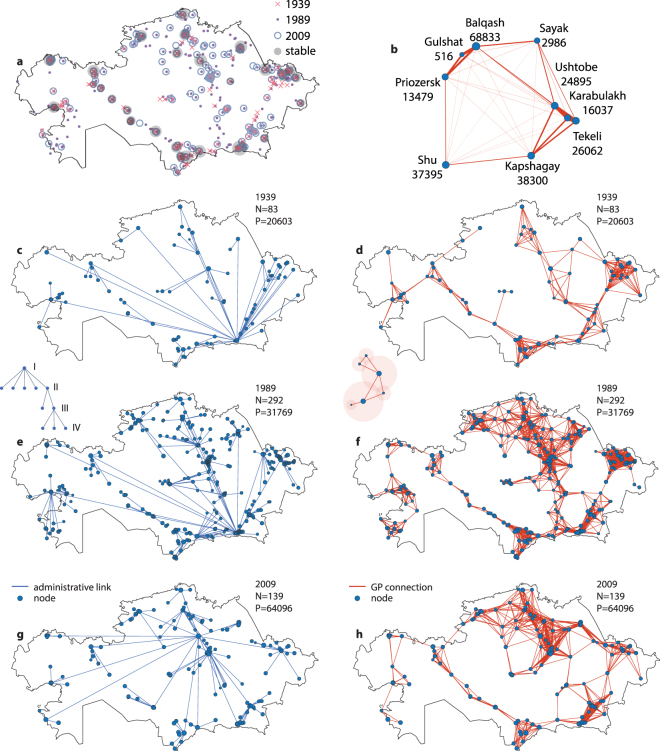
The urban networks. (**a**) Coordinates of the cities on the geographical map for different time points (see legend for color codes). The grey points (whose size is proportional to the population averaged over time) correspond to those cities, which were registered in all censuses. (**b**) The vizualization of link weights in GPN between several cities in southeastern region, 2009. The width of the line connectiong two cities is proportional to the weight of the link between them. (**c**,**e**,**g**) Administrative networks created using the data of the 1939, 1989 and 2009 census. The links represent the subordinative relations between cities belonging to neighbouring levels of hierarchy, as shown in inset. (**d**,**f**,**h**) Geo-population network constructed using the data of the 1939, 1989 and 2009 census. The inset roughly shows the principle of GPN connections. *N* and *P* stand for number of nodes and the average city population in the given year, respectively. The maps were prepared using MATLAB software.

To get a visual representation on how the network evolves, the coordinates of the cities are shown in Fig. 1a for several censuses. The growth and reduction of the number of cities involve all parts of the network. Yet, some areas on the map are revealed to be historical hubs, preserving urbanization at all time points. These “stable” cities (shown by grey) are mostly the regional centers and former capitals of the country, and their location is the result of a complicated interplay of historical, geographical and climat factors. For instance, some of the stable regions are located far from the big urban clusters, and correspond to towns with relatively small populations. The nature of their viability is accounted by the transport/trade paths (such as the silk way, which was historically passed through them)^20^.

## Results

Today, it is obvious that the administrative division of cities plays a crucial role in distribution of funds and resources. To reveal the hierarchy of cities corresponding to each time point, we have used the information of their administrative relations. We are considering only cities and towns, which are divided into three (or four, depending on a census) levels of hierarchy (including the capital), depending on their administrative role and importance. The number of cities on each level of the hierarchy is reported in Table 1 for all censuses.

Each city, regardless of the level of hierarchy to which it belongs, is represented by a network’s node in our study. The links represent the connections between neighbouring levels of hierarchy: if the city *i* is subordinated to (i.e. in the authority of) the city *j*, then a undirected connection between node *i* and *j* exists in the graph (see Methods, and Fig. 5 for details). The structures of the hierarchical administrative network (HAN) for the 1939, 1989 and 2009 census is depicted in Fig. 1c,e,g [other visualizations of HAN can be seen in Fig. 1 in the Supplementary Material]. The networks describing the 1939 and 2009 year are much more simple, then the one of 1989. However, they have completely different structures and a different localization of the main capital node. The number of cities in each level drastically increases until the eighties, but the second level (the regional centers) becomes almost stable already in the sixties (see Table 1), and the further growth is mostly due to the emergence of small cities (third and fourth levels). However, with the dissolution of Soviet Union in 1992, the nineties saw revolutionary changes in the administrative rules and regulations. In particular, two points are remarkable here: (i) almost half of the regional centers lost their statuses, and were classified as belonging to the third hierarchical level; (ii) the two lower levels (the third and fourth ones) were merged together, resulting in a sharp increase of the number of cities belonging to the third level. The centralization of the 2009 network due to the elimination of the fourth hierarchy level can be seen by the naked eye.

However, administrative relationships are not the only factor determining the interaction between cities and towns^21^, which is connected also with their geographic location, determining the mobility of citizens from one town to another, economical and trade relations. The spatial clustering of cities and the emergence of the historical hubs of urbanization seen in Fig. 1a refers us to the concept, that the probability of emergence of a new city is higher in the geographical proximity of already existing ones^22^. It gives us reason to suggest, that we can still try to quantify the connections between cities using the information on their coordinates in the way similar to correlated percolation model^23,24^. To improve the accuracy of our estimations we also taking into account the populations of cities, supposing that ties between large cities is stronger, then between small ones^25,26^. We suppose that the strength of the connection between two cities weakens with the distance between them, but the increase in the city population literally extends its area of influence, i.e. its effective connectivity range. The Fig. 1b shows the principle of network construction (see Methods for the details), where we have depicted several cities and weighted links between them. The figure reveals clusterization of cities, which are located close to each over. Besides that, large cities spread connections between each over on much longer distances, while cities with small populations have connection only to nearest neighbors.

In Fig. 1d,f,h we represent the structure of the geo-population network (GPN) in the 1939, 1989 and 2009 years [visualizations for the other censuses are available in Fig. 1 in the Supplementary Materials], which displays the topology of a spatially-distributed scale-free network (see Methods for the detailed GPN properties), with a modular structure made of the number of interconnected clusters. Comparison of the obtained structure with the map in Fig. 1a allows to highlight that GPN fits closely the spatial clustering of the cities, which are strongly connected in the GPN. This accounts for cooperation between the cities and towns located in each common region. Moreover, we can track the evolution of the given network over the seventy years and compare it with the changes in the administrative structure. In the 1939 the south-eastern region of the country is dominated by the number of nodes and connections between them, containing the capital of the country. But one can track the gradual strengthening of the northern region of the country until the 1989 due to specific geopolitical processes [see Figs 1c–h and 1 in the Supplementary Materials]. While in the 1989 the capital is still in the southern region (Alma-Ata), the dissolution of the Soviet Union is followed by the transfer of the capital to the northern region (Akmola city; renamed as Astana since 1992), that is directly connected to the observed trends.

But our main goal is describe the instantaneous interplay between HAN and GPN beyond the study of their main topological features separately. For this task we consider them in the framework of multiplex network^27^: each city on the hierarchical layer is connected to itself on the geo-population layer. The multiplex adjacency matrix is obtained as:

1 $${w}^{M}=[\begin{array}{cc}{w}^{{\rm{HAN}}} & E\\ E & {w}^{{\rm{GPN}}}\end{array}],$$

where *w* ^HAN^ and *w* ^GPN^ are the adjacency matrices of administrative and geo-population layers, respectively, and *E* is the identical matrix of size *N*. Such multilayer analysis reveals how features in one layer directly influence those of the other layer^28^. In particular, we consider two types of centralities, eigenvector and betweenness, which constitute very relevant measures in spatially-distributed urban networks^29^, as they are strongly connected with information and agent spreading in the networks (see Methods for details). In the context of urban networks, these measurements may reveal the diffusion of funds, people migration and natural resources between cities^30^.

**Figure 2:**
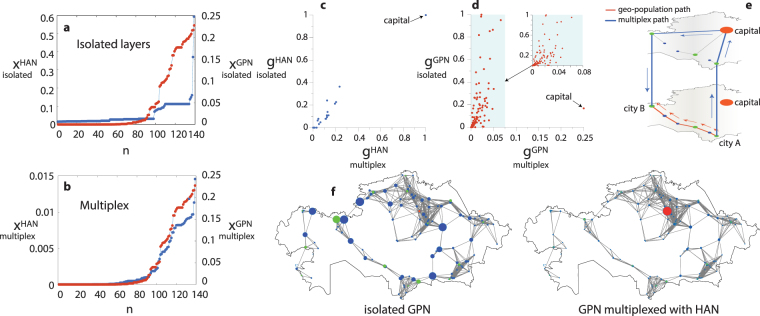
Centralities of the urban networks. (**a**,**b**) Sorted values of eigenvector centrality of the nodes belonging to the administrative network, *x* ^HAN^, and to the GPN, *x* ^GPN^. Values are calculated for two cases: while the layers are isolated (**a**) and arranged in a multiplex network (**b**). Values corresponding to the administrative network, *x* ^*h*^, are shown by blue, while red refer to geo-population network, *x* ^GPN^. (**c**,**d**) Betweenness centrality of each node in the multiplex network versus its value in the isolated layer for (**c**) administrative network, and (**d**) geo-population network. (**e**) The evolution of shortest paths with multiplexing: orange lines depict the shortest path between city A and city B in isolated geo-population network (lower plot), while blue lines show the shortest path between this cities in multiplex network, which is going through the administrative layer (upper plot). (**f**) Visualization of isolated and multiplexed layers. The size of the node is proportional to its betweenness centrality. Orange color refer to capital, green and blue to regional centers and regular cities respectively. All presented data correspond to the census of 2009. The maps were prepared using MATLAB software.

We start with eigenvector centrality, and calculate its value for each node in both HAN and GPN. Values are calculated for two cases: (i) when the layers are isolated and (ii) when they are arranged in a multiplex network. The case (ii) implies that we calculate the eigenvector centrality of nodes in a network of size 2 × *N* and then separate the values corresponding to the HAN and GPN layer. Further, we sort the obtained values for both cases (i) and (ii) and plot them in Fig. 2a,b, respectively. Such representation allows us to encapsulate the structural partition of the layers, while they are isolated (see Fig. 2(a)) and multiplexed (see Fig. 2(b)). While the centrality of the isolated HAN (blue dots) features several stages, which are correlated with the levels of hierarchy, the GPN (red dots) is characterized by a more complex, continuous structure of node centralities. When multiplexed, the node centrality of the administrative layer drastically changes. The result is consistent with reported in ref.^31^: when two initially separated networks are connected by a link, the one of them featuring the higher algebraic connectivity^32^ obtains the majority of eigenvector centrality. Here, however, an additional phenomenon is observed: the centrality vector of the HAN becomes very similar to that of the GPN. This means that nodes in the hierarchial administrative layer not only loose their centrality, but also acquire the ranking of the other layer. The conclusion is that GPN is dominant, and determines the relevance of a given city. If one examines the correlation between the centrality of each node for multiplexed and isolated layer (see Fig. 2 in the SM), one actually finds that only the capital and one of most developing cities (Karaganda) exhibit a direct correlation of the eigenvector centrality in the HAN, whereas the centrality of GPN nodes is practically independent on whether the layers are or not connected.

Next we consider the betweenness centrality. Once again, we calculate each node betweenness for both cases of (i) isolated and (ii) multiplexed networks. Figure 2c,d show the betweenness of each node in isolated layer versus its value when the layers are interconnected for both HAN or GPN. Here, one observes the reverse process. The node betweenness of the isolated administrative layer exhibits a straight correlation with its betweenness in multiplex network, and practically does not change its absolute value. At the same time, geo-population network dramatically (except for the capital) shrinks all values of betweenness in the process of multiplexing. Notice the large gap between the capital and other cities in both the administrative (c) and geo-population (d) layers. In GPN the capital node has a moderate value of betweenness in the isolated case, but became the most central node as soon as the layers are connected, with an absolute value of betweenness growing even besides the isolated case. This fact implies that the spatially-dependent GPN not simply looses its betweenness, but rearranges its pathways according to the HAN. This is illustrated in Fig. 2e, where we show how the shortest path between city A (Kyzylorda) and B (Aktobe) (both regional centers) in geo-population layer evolves with multiplexing. The shortest path in isolated layer (orange links and arrows) takes five steps, while multiplexing allows to reduce the number of steps to four (blue arrows and blue lines), passing through the capital on the administrative layer.

**Figure 3:**
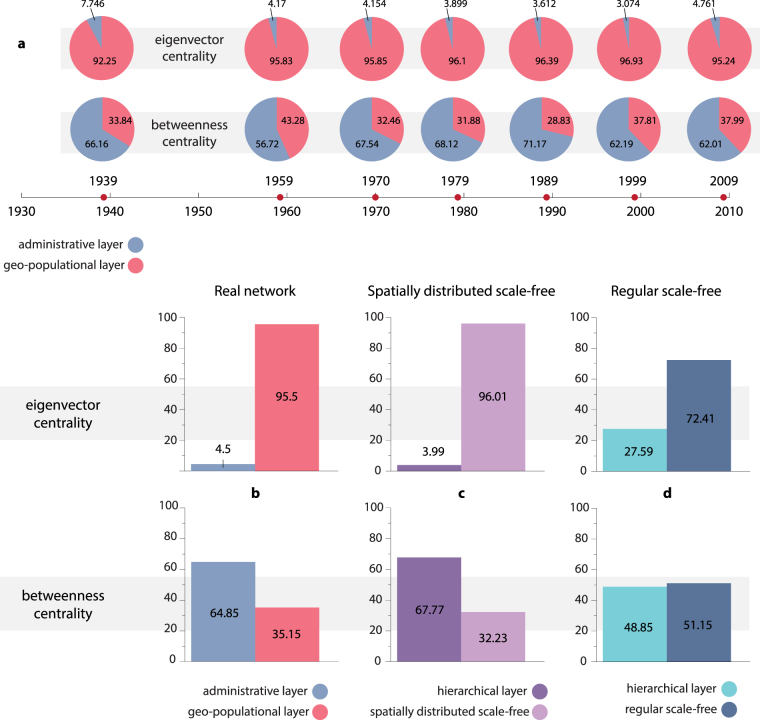
Total centrality of layers in the multiplex network. (**a**) The time evolution of the integral value of each type of centrality of the nodes belonging to administrative and geo-population layer, which are arranged in multiplex network. The values of the eigenvector (upper row) and betweenness centrality (lower row) are shown in percentage. (**b**–**d**) Total centrality in each layer for the real network, for spatially-distributed scale-free and for a regular scale-free network. The real data (**b**) are averaged over the 7 available datasets, numerically obtained values (**c**,**d**) are averaged over 10^3^ independent realizations of the networks.

In order to better visualize the redistribution of shortest paths, the size of the nodes reported in Fig. 3f accounts for their betweenness in the geo-population layer, when it is considered to be isolated (left) and multiplexed (right) with the administrative layer. In the isolated GPN, the larger values of betweenness correspond to ordinary cities, which are located outside the urban clusters and play the role of the paths between them, while the capital and most of the regional centers exhibit low values of betweenness. However, when the geo-population and administrative layers are multiplexed, the distribution of betweenness becomes similar to that of the hierarchical network, producing an effect which is the inverse of the observed with eigenvector centrality.

As mentioned above, the system under study underwent various structural changes over the considered years. To reveal if there are temporal changes of the observed effects we calculate betweennes and eigenvector centrality of the nodes in different time points only for multiplexed layers. Further, we separately calculate the integral value ${u}_{i}^{{\rm{MN}}}$ of betweenness and eigenvector centrality for the nodes belonging to different layers:

2 $${u}_{T}^{{\rm{HAN}}}=\sum _{i=1}^{N}\,{u}_{i}^{{\rm{MN}}},$$

3 $${u}_{T}^{{\rm{GPN}}}=\sum _{i=N+1}^{2N}\,{u}_{i}^{{\rm{MN}}}\mathrm{.}$$

Such quantities are reported (in percentage, and assuming that ${u}_{T}^{{\rm{HAN}}}+{u}_{T}^{{\rm{GPN}}}=\mathrm{100 \% }$) in Fig. 3a. It becomes apparent that at all times the geo-population layer obtains the majority of the eigenvector centrality, which takes 95.5% on average. The betweenness centrality demonstrates more pronounced changes in time. However, this changes are more likely fluctuations around its mean value, which is 65% for the administrative layer. The latter implies that despite the redistribution of the shortest paths in the network the sufficient amount of shortest paths (35% on average) still goes through GPN. Summarizing the above, we have revealed the sustainability of total centrality of the layers for the given urban network.

To prove that our results are not associated with spurious features of the considered data, we produce and analyze various numerically produced geo-population (spatially-distributed scale-free networks) and hierarchical networks (see Methods for the details of the algorithm used for the network construction). Furthermore, we compare the obtained result with those for the regular spatially-independent scale-free network multiplexed with hierarchical structures. The total eigenvector and betweenness centrality of each multiplexed layer in Fig. 3 for the real (b), spatially-distributed scale-free network (c) and regular scale-free network (d). As the model networks are created using randomization of initial conditions, we have aggregated the results obtained over 10^3^ independent realizations of both spatially-distributed and regular scale free networks. At the same time, we average the real values over the seven available time points. One can see that, despite the differences in the amount of data (7 real vs. 1,000 model datasets), the spatially-dependent scale-free network (c) accurately reproduces the same relationships emergent in the real data (b).

The latter result enables us to claim that the observed effect is a quite universal feature of the multiplex interaction betweeen hierarchical and spatially distributed network. When, indeed, a regular (i.e. spatially independent) scale-free network is considered (Fig. 3d), the scenario drastically changes: now the hierarchical network obtains around 30% of the eigenvector centrality, that is much more than in the spatially-dependent case. At the same time, the total betweenness centrality of scale-free layer is almost equal to that of the hierarchical layer, i.e. the effect observed in spatially-distributed networks completely disappears. Such weakening of the hierarchical layer is caused by emergence of direct connections between structural hubs, which aggregate the majority of betweenness, while in real networks these hubs have no direct geo-population connections. Hence, the observed phenomena refers to the genuinely spatial nature of real urban networks.

**Figure 4:**
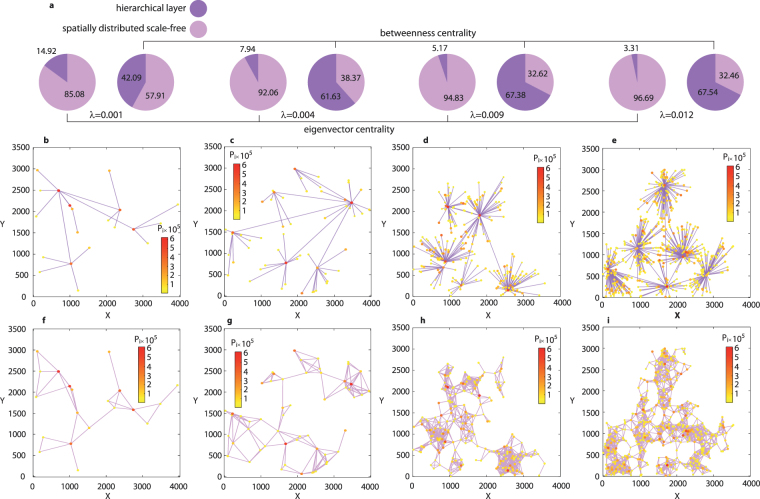
Topological features of the urban network. (**a**) Total centrality in each layer for a spatially distributed scale-free network and a hierarchical layer arranged in a multiplex network, at different values of the density gradient *λ*. (**b**–**i**) The corresponding visualisations of the hierarchical layer (**b–e**) and of the spatially distributed scale-free network (**f**–**i**): (**b**,**f**) *λ* = 0.001; (**c**,**g**) *λ* = 0.004; (**d**,**h**) *λ* = 0.009; (**e**,**i**) *λ* = 0.012. The visualizations were prepared using MATLAB software.

Let us examine how the density gradient, *λ*, effects the relation of centralities in the multiplex network. Figure 4a shows the centrality diagrams for the model spatially-distributed networks multiplexed with hierarchical structures at several values of *λ*, while Fig. 4b–i depict the corresponding spatial visualizations. When the density gradient is small (*λ* = 0.001; Fig. 4b,f), the probability of emergence of the new nodes is weakly correlated with the location of the existing ones. As a result, the network rapidly becomes a connected graph (see Methods for the steps of the numerical modeling). Due to the simplicity of the resulting structure, the relationship of centralities in this case is closer to that of a regular scale-free network. The increase of *λ* (Fig. 4c,g) leads to aggregation of new nodes around the initial ones, and the overall network structure becomes modular. Here, the relation of centralities (*λ* = 0.004) drastically changes, and becomes very similar to the real case: the hierarchical layer obtains the majority of betweenness, while spatially-distributed network takes >90% of eigenvector centrality. A further increase of the density gradient (*λ* = 0.009, Fig. 4d,h, and *λ* = 0.012, Fig. 4e,i) results in the growth of the node density in the modular structures. However, the centrality relation changes only weakly, and in all cases it remains close to the real network case. We can therefore conclude that the proposed model nicely reproduces the effect under study for values of *λ* larger than 0.004.

The value of the density gradient effects the properties of the networks build on real data in the same fashion. The centrality dependencies are shown in Fig. 3 in the Supplementary Material, together with the illustration of the networks structures. Basically, there are three regions of *λ*. The lowest values (*λ* < 0.0015) yield non-physical global coupling in the network. The range 0.0015 < *λ* < 0.005 corresponds to a transient region, where the network starts to feature modularity. In the third region of large values of *λ* > 0.005 the structure becomes stable and the centrality properties are practically not affected by the value of *λ*. While constructing the networks based on real data we work with value of density gradient, which belongs the the third region (*λ* = 0.009), corresponding to the stable structure after the transient process.

## Discussion

Summing up our results, we have revealed two important effects which occur in the urban networks when both administrative and geographical relations between its cities are taken into account in a multiplex framework: (i) the geo-population network aggregates eigenvector centrality and imposes its own ranking to the administrative (hierarchical) network, (ii) the administrative network connects the structural hubs and redistributes the shortest paths between nodes in the geo-population layer.

We also revealed the relationship between the total values of centrality in each layer, which was fully supported by numerical simulations. The comparison with the regular scale-free network reveals that the observed effects are intimately connected with the spatially distributed nature of the real network. More specifically, the effect of redistribution of shortest paths refers to the absence of direct connections between the main hubs of the urban network. A similar comparison between spatially extended and non-spatially extended scale-free networks was made earlier in ref.^33^, where the Authors have shown that node betweenness centrality and the node degree are significantly less correlated if the structural hubs have no direct connections. Our study is in a good agreement with this result, because (i) structural hubs (high-degree nodes) of the geo-population network exhibit low betweenness and (ii) the synthetic case of a regular scale-free network leads to the disappearance of the observed effects.

Let us speculate also on the practical implications of our results. The urban network under study displays a complex modular structure, where clusters are connected between each other by a small number of nodes. Obviously, these nodes are of crucial importance for the country, and their prosperity is connected with the development of the transportation network, trading, etc. On the other hand, the administrative network is responsible for the distribution and diffusion of funds and innovations inside the country from the upper levels^34,35^. That’s why one of the presumptive ways to secure their foothold is to transfer them on the upper level of hierarchy. Notably, the inverse politics took place in the Republic of Kazakhstan: many of regional centers and regular cities lost their statuses in 1990s. This, in turn, led to the extinction of the city network (as it is seen from the data in Table 1), a phenomenon which was also reported by recent publications in the area^36,37^.

There are other global examples, that could be described by multiplex interactions between spatially-distributed networks (which are formed by historical and geographical factors) and spatially-independent (not necessarily hierarchical) networks. The most vivid and, somehow, topical case is the interplay between the geography of countries (which have their own historically conditioned culture and ideology), and the World Wide Web (WWW) network, which actually introduce connections between culturally inhomogeneous areas. This, in turn, leads to a variety of reactions on the side of the geographical layers: from the active integration of the spatially-independent layer^38^, to the actual cutting off or filtering of the inter-layer connections^39^. There is no doubt, therefore, that further investigations of the interplay between spatially-distributed and spatially-independent networks will enable our community to predict and control the behavior of many real world systems.

## Methods

### Administrative network

**Figure 5:**
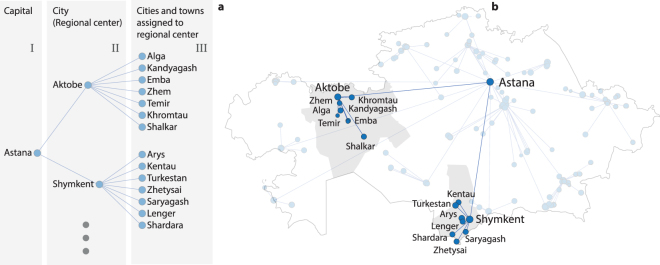
Construction of the administrative network. (**a**) The schematic representation of the administrative hierarchy of cities in two regional centers and (**b**) the visualization of the corresponding part of the network on the map. The data correspond to the 2009 census. The visualization was prepared using MATLAB software.

The schematic table showing the administrative hierarchy of the cities for two country regions of the 2009 census is depicted in Fig. 5a. We are considering only cities and towns, which belong to three (as depicted in the example) or four levels of hierarchy depending on the year. The Republic of Kazakhstan is divided into regions, each of which contains a principal city, which is the regional center (or capital of the region). Each regional center (II level) is in the authority of the State’s capital (I level). The III level contains other cities and towns located in the regions, each of them being in the authority of the corresponding regional center. One easily notices that, before 1999, there are also small towns belonging to the fourth layer of hierarchy. In our study we consider the relations between levels of hierarchy as undirected connections, as illustrated by the blue lines in Fig. 5a. In order to construct the adjacency matrix of the administrative layer, we suppose that if the city *i* is in the authority of the city *j*, then a undirected link, ${w}_{ij}^{{\rm{HAN}}}={w}_{ji}^{{\rm{HAN}}}=1$, is formed. The visualization of the graph is shown in Fig. 5b for two typical regions.

### Geo-population network

We use the populations and coordinates of the cities to construct the GPN. The distribution of city populations (see Fig. 4 in the Supplementary Information) fits a power law $N({P}_{i})\propto {P}_{i}^{-\gamma }$, where *γ* = 1.8, which is very close to the typical *γ* = 2.0 characterizing urban systems and the Zipf’s law^40^. The geo-population network is built by using the principles of the gravity^25^ and correlated percolation^23,24^ models. First, one determines the weight of the link, ${w}_{ij}^{{\rm{GPN}}}$, between each pair of cities, for a given time slice:

4 $${w}_{ij}^{{\rm{GPN}}}={w}_{ji}^{{\rm{GPN}}}=l{\rm{g}}\,{P}_{i}\,{\rm{lg}}\,{P}_{j}\,{e}^{-\lambda {R}_{ij}},$$

where *P*_*i*_ and *P*_*j*_ are the populations of the cities *i* and *j*, respectively, *R*_*ij*_ is the Eucledian distance between them and *λ* is the density gradient. The density gradient (reflecting the urban spreading) was chosen to be *λ* = 0.009, according to ref.^22^ and our calculations (see Fig. 3 of Supplementary Materials). If one plots the weight distribution (see Fig. 4 in the SM), most of it is fitted by a power law, except the the first bin which displays a value almost 10 times larger than expected. Since the administrative network is binary, we need (for consistency) to binarize the GPN as well. To this aim, we first prune the links sequentially starting from the weakest ones, until the network became disconnected. At that point, we restore the weight of the last removed link, *w*_*threshold*_, and set all remaining weights equal to unity. Remarkably, the obtained threshold value *w*_*threshold*_ reveals that the manipulations accurately remove negligibly small weights (as it is seen in Fig. 4 of the SM), and the obtained structure displays the topology of a spatially-distributed scale-free network^41^.

### Centrality measures

The eigenvector centrality measures the importance (or ranking) of a node in a given network, by accounting not only for its number of connections, but also (in an iterative way) for the relevance of its neighbours^42^. The definition is:

5 $${x}_{i}=\frac{1}{\nu }\,\sum _{j=1}^{N}\,{w}_{ij}{x}_{j},$$

where *ν* is here the largest eigenvalue of the adjacency matrix, and the *x* is its corresponding eigenvector. The betweenness centrality of a node quantifies the number of shortest paths in the network which are passing through it^30^. Its definition is:

6 $${g}_{i}=\sum _{i\ne j\ne k}\,\frac{{\sigma }_{jk}^{i}}{{\sigma }_{jk}},$$

where ${\sigma }_{jk}^{i}$ is the number of shortest paths between node *j* and *k* which pass through node *i*, and the *σ*_*jk*_ is the total number of shortest paths between *j* and *k*. During the calculation, the measure is conveniently normalized in the interval [0:1].

### Model spatially-distributed network

The numerically produced spatially-distributed network consists of two layers, each one containing an identical set of nodes. Each node *i* represents a city which is labeled by three numerical features, which we need to obtain: the X and Y coordinates on the geographical space of size 4,000 × 3,000, and the population *P*_*i*_. In the real urban systems, the cities are not located randomly, and there is a dependence for the appearance of new nodes: a new city will probably appear in the vicinity of an existing one. To conform with this principle, we use the modified correlated percolation model^22^, which assumes that the probability of appearance of a new city *i* depends on the occupancy of the neighborhood.

First, we select the initial set of *N*_12_ nodes and randomly assign a) their XY coordinates, and b) their population by picking a value in the range from 10^5^ to 5 × 10^5^. The value *N*_12_ determines the number of nodes assigned to the first (always one) and the second (*N*_12_ − 1) level in the hierarchical administrative network, and was chosen as *N*_12_ = 6 in this study. However, we found that variation of *N*_12_ in the range $[5,30]$ affects only weakly the results. Next, a temporary adjacency matrix *W* of *N* × *N* (*N* = *N*_12_) is created, and filled with zeros.

After that, one starts to grow the synthetic urban network, and start adding new nodes (cities) in the following way:

(i) The temporary adjacency matrix *W* acquires a new (*N* + 1)-th column and row, which are filled with zeroes ($\forall j$, *W*_(*N*+1)*j*_ = *W*_*j*(*N*+1)_ = 0).
(ii) One randomly assigns coordinates of the node (*N* + 1) and determines the probability of connection to each of the already existing nodes:
  7 $${p}_{(N+\mathrm{1)}j}={e}^{-\lambda {R}_{(N+\mathrm{1)}j}},$$
  where *R*_(*N*+1)*j*_ is the distance between nodes (*N* + 1) and *j* and *λ* = 0.009 is (once again) the density gradient.
(iii) For each pair (*N* + 1) ≠ *j* one sets *W*_(*N*+1)*j*_ = 1 if the uncorrelated random number with a uniform probability distribution [0, 1] is lower than *p*_(*N*+1)*j*_.
(iv) If the new node (*N* + 1) has at least one connection $({\sum }_{(N+\mathrm{1)}\ne j}\,{W}_{(N+\mathrm{1)}j} > 0)$, this node remains in the network, and the number of network nodes is increased by 1: *N* = *N* + 1. Overwise, one returns to step (ii).
(v) One randomly assigns the population of the new node, *P*_*N*_, from a probability distribution $\sigma ({P}_{N})={P}_{N}^{-1}$ in the range [10^3^, 3 × 10^5^]. At the same time, each population *P*_*j*_, for which *W*_*Nj*_ = 1, obtains additional 10% of the *P*_*N*_.

After the appearance of each new node, one determines if the adjacency matrix corresponds to a connected graph. If not, one continues adding nodes until the condition is met. When the updated adjacency matrix, *W*_*ij*_, becomes a connected graph, we then work only with the final values of node populations and coordinates. It should be noted that, due to the algorithm, the final number of nodes, *N*, can sufficiently vary in independent realizations, but generally it lies in the range from 300 to 400 nodes (for the given size of the geographical space, and the given *λ* = 0.009). The adaptive growth of the cities (v) leads to formation of a real-like distribution of populations, which fits a law $N({P}_{i})\propto {P}_{i}^{-\gamma }$, where *γ* takes a value close to 2^40^, as in the real urban systems. The distribution of distances, *R*_*ij*_, also displays a dependence very similar to the real case (see Fig. 5 of the SM).

The spatially-distributed scale-free (geo-population) network is constructed using the values of populations and distances between nodes via the same method as the real network (Eq. (4)). The numerically obtained weight distribution and threshold value (*w*_*threshold*_) reveal that the reduction of the connections eliminates only negligibly small weights, which does not fit the power law (once again, see details in Fig. 5 of the SM).

To set a hierarchical administrative network, one picks the node with the largest population from the initial set of *N*_12_, and assigns it the role of “capital city”. The other (*N*_12_ − 1) (5 in this study) nodes from the initial set are assigned to the second level of hierarchy, and have connection with the capital node. Each remaining node of the network is set to belong to the third level with nodes number *N*_3_ = *N* − *N*_12_, and is connected to the spatially-nearest upper-level nodes.

### Model spatially-independent network

The spatially independent network is obtained by Barabasi-Albert (BA) model^43^, and then multiplexed with a hierarchical layer. As in the case of spatially-dependent networks, one initially chooses a set of six cities, which became the “capital” and the second level nodes, while and the remaining nodes are randomly assigned to the upper-level nodes. The size of each network was chosen randomly in range [300:400] nodes.

## Electronic supplementary material

Supplementary File
