# Supplementary material for: Interplay between geo-population factors and hierarchy of cities in multilayer urban networks
Source: Sci Rep. 2017 Dec 8;7:17246. doi: 10.1038/s41598-017-17576-8 (PMC5722944; doi:10.1038/s41598-017-17576-8)
Supplement: Supplementary file 1 — Supplementary File [file 41598_2017_17576_MOESM1_ESM.pdf]

Supplementary Material:

Interplay between geo-population factors and hierarchy of cities in multilayer urban networks

Vladimir V. Makarov, Alexander E. Hramov, Mikhail V. Goremyko, Daniil Kirsanov, Vladimir A. Maksimenko, Alexey V. Ivanov, Ivan A. Yashkov, Stefano Boccaletti

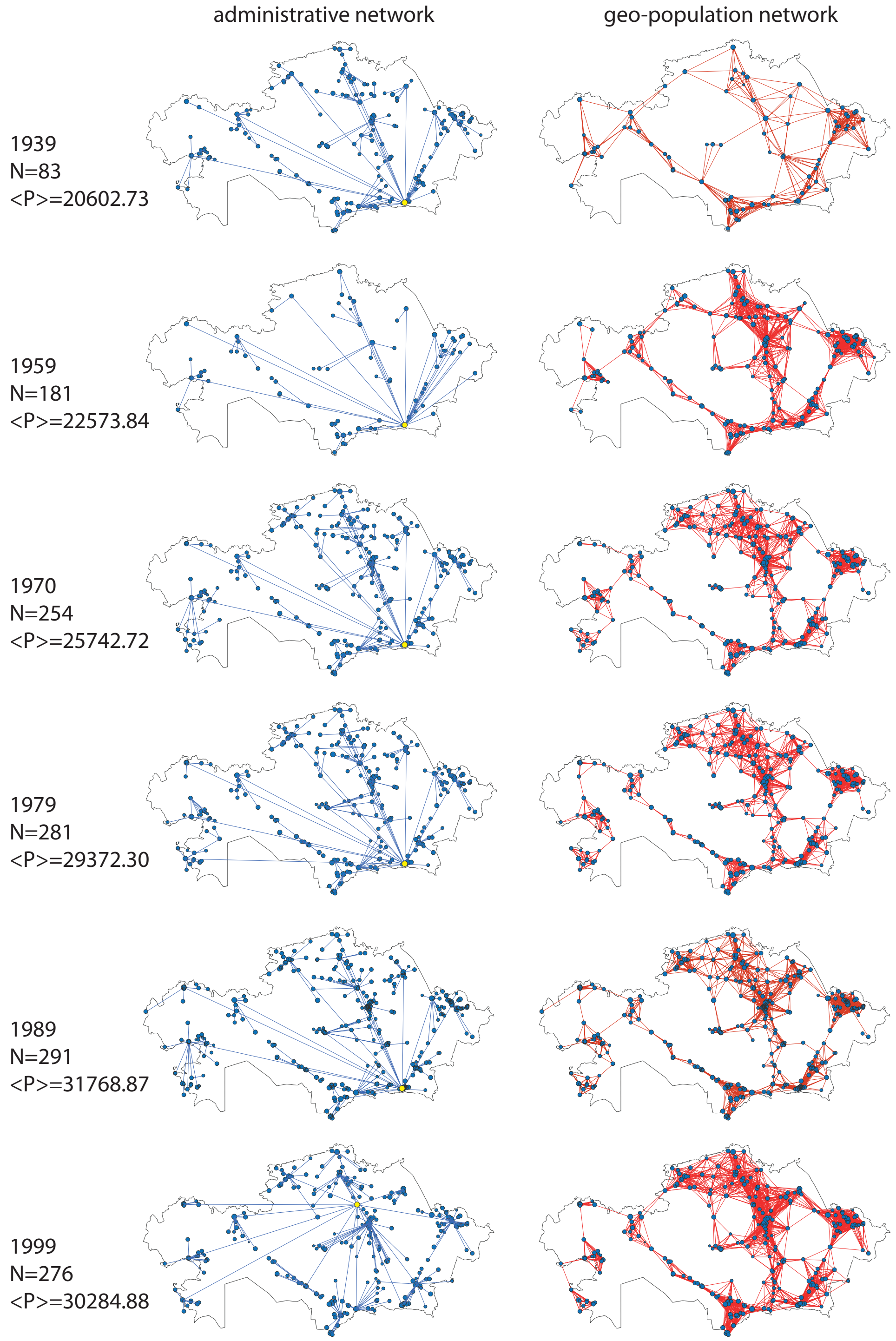

FIG. 1. (Color online). Visualizations of the maps of the administrative and geo-population networks at different years. The capital city is always shown in the administrative networks by a yellow dot. N and $< P >$ denote the number of cities and average urban population, respectively. The maps were prepared using MATLAB software

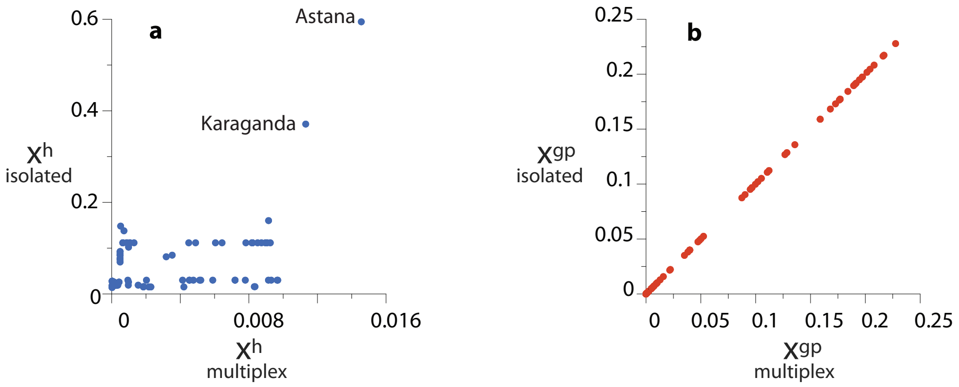

FIG. 2. (Color online). Eigenvector centrality for each node of the multiplex network (horizontal axis) versus its value in isolated layers (vertical axis) for (**a**) the administrative network and (**b**) the geo-population network. Data correspond to the 2009 census

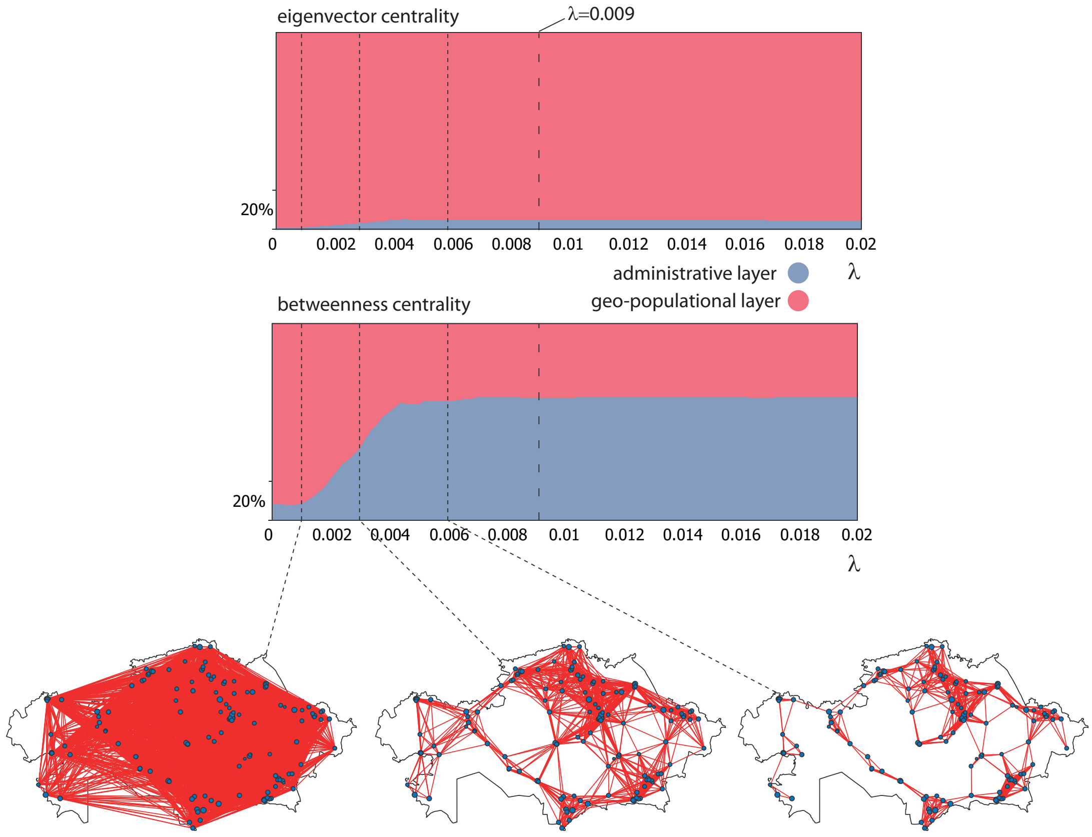

FIG. 3. (Color online). The relation between centralities of the netwroks build using the real data versus the density gradient, λ.

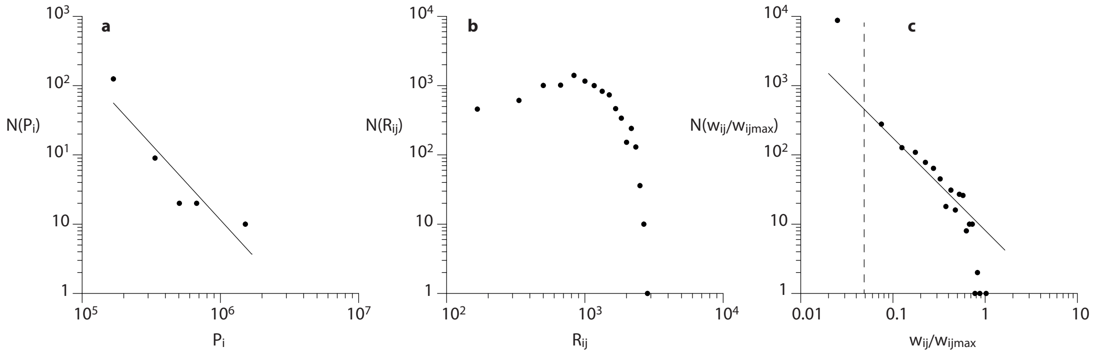

FIG. 4. (Color online). The geo-population network. **a** Distribution of city populations Pi (dots) and its approximation with a power law $N(P_i) = P_i^{-\gamma}$, where $\gamma = 1.8$ (solid line). **b** Distribution of the distances R_{ij}. **c** Weight distribution (dots) and its power law approximation with exponent $\gamma = 1.5$ (solid line). Weights are normalized to w_{ijmax}, i.e. to the maximal weight value. The minimal weight, $w_{treshhold}$, is shown by a vertical dashed line.

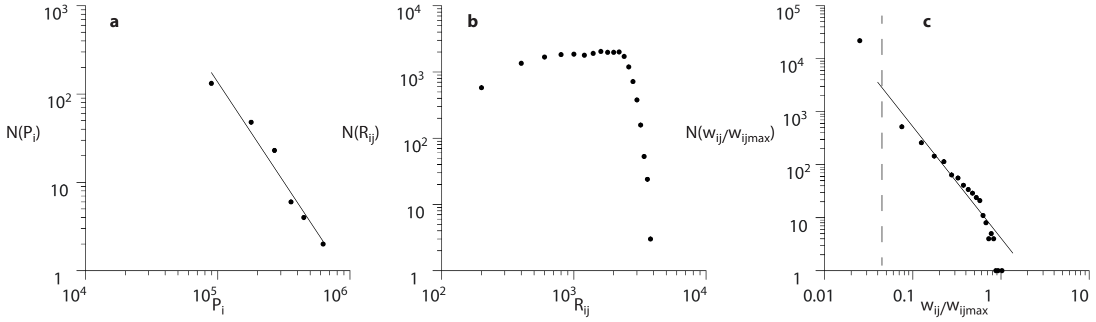

FIG. 5. (Color online). The model spatially-distributed network. The data reported in all panels of the Figure correspond to the same realization of the network. **a** Distribution of city populations P_i (dots) and the approximated power law $N(P_i) = P_i^{-\gamma}$, with $\gamma = 2.26$ (solid line). **b** Distribution of distances R_{ij}. **c** Weight distribution (dots) and power law approximation, with exponent $\gamma = 2.0$ (solid line).
